# The Role of European Starlings (*Sturnus vulgaris*) in the Dissemination of Multidrug-Resistant *Escherichia coli* among Concentrated Animal Feeding Operations

**DOI:** 10.1038/s41598-020-64544-w

**Published:** 2020-05-15

**Authors:** Jeffrey C. Chandler, Jennifer E. Anders, Nicolas A. Blouin, James C. Carlson, Jeffrey T. LeJeune, Lawrence D. Goodridge, Baolin Wang, Leslie A. Day, Anna M. Mangan, Dustin A. Reid, Shannon M. Coleman, Matthew W. Hopken, Bledar Bisha

**Affiliations:** 1 U.S. Department of Agriculture, National Wildlife Research Center, Fort Collins, CO USA; 2 University of Wyoming, Department of Animal Science, Laramie, WY USA; 3 University of Wyoming, Department of Molecular Biology, Laramie, WY USA; 4 Food and Agriculture Organization of the United Nations, Rome, Italy; 5 University of Guelph, Food Science Department, Guelph, Ontario Canada; 6 Iowa State University, Department of Food Science and Human Nutrition, Ames, IA USA; 7 Colorado State University, Department of Microbiology, Immunology, and Pathology, Fort Collins, CO USA

**Keywords:** Antimicrobial resistance, Bacteriology, Invasive species

## Abstract

Antimicrobial use in livestock production is a driver for the development and proliferation of antimicrobial resistance (AMR). Wildlife interactions with livestock, acquiring associated AMR bacteria and genes, and wildlife’s subsequent dispersal across the landscape are hypothesized to play an important role in the ecology of AMR. Here, we examined priority AMR phenotypes and genotypes of *Escherichia coli* isolated from the gastrointestinal tracts of European starlings (*Sturnus vulgaris*) found on concentrated animal feeding operations (CAFOs). European starlings may be present in high numbers on CAFOs (>100,000 birds), interact with urban environments, and can migrate distances exceeding 1,500 km in North America. In this study, 1,477 European starlings from 31 feedlots in five U.S. states were sampled for *E. coli* resistant to third generation cephalosporins (3G-C) and fluoroquinolones. The prevalence of 3G-C and fluoroquinolone-resistant *E. coli* was 4% and 10%, respectively. Multidrug resistance in the *E. coli* isolates collected (*n* = 236) was common, with the majority of isolates displaying resistance to six or more classes of antibiotics. Genetic analyses of a subset of these isolates identified 94 genes putatively contributing to AMR, including seven class A and C β-lactamases as well as mutations in *gyrA* and *parC* recognized to confer resistance to quinolones. Phylogenetic and subtyping assessments showed that highly similar isolates (≥99.4% shared core genome, ≥99.6% shared coding sequence) with priority AMR were found in birds on feedlots separated by distances exceeding 150 km, suggesting that European starlings could be involved in the interstate dissemination of priority AMR bacteria.

## Introduction

Antibiotic use in animal agriculture for prophylaxis, therapy, and growth promotion is generally recognized to coincide with the proliferation of antimicrobial resistance (AMR) in associated bacterial communities^1–3^. This is problematic not only for animal agriculture, but also because many antibiotics used in livestock are identical in structure or function to those used in human medicine^4,5^. Furthermore, the use of antibiotics in food animals is predicted to increase globally for the foreseeable future and is expected to exacerbate the AMR problem^6^.

AMR development and circulation in livestock systems and the subsequent transmission of AMR bacteria and genes to humans is a complex dynamic with numerous inputs and outflows, often framed as part of the OneHealth continuum with direct and indirect costs to health, the environment, and the economy^7,8^. While certain environmental inputs, including human, animal, and manufacturing waste, have received significant attention in promoting AMR, less is known about the contributions of other ecological pathways, including wildlife incursions and dispersal^9^. Nonetheless, it is recognized that wildlife, such as rodents, birds, and mesocarnivores that frequent animal production facilities, harbor AMR bacteria with similar AMR phenotypes and genotypes to those found in bacteria from associated livestock^10–14^.

The involvement of wild birds in the maintenance and dissemination of AMR across agricultural landscapes is particularly intriguing due to the ability of birds, especially migratory birds, to transport and shed bacteria in their feces over large distances^15^. Frequent carriage of AMR bacteria and AMR genes has been reported for multiple species of wild birds^12,14,16–20^. Thus, bird species that are in frequent contact with anthropogenic foci of AMR, such as livestock production, may play an important role in the dissemination and propagation of AMR across the landscape.

European starlings *(Sturnus vulgaris*) are peridomestic birds that are invasive agricultural pests in the United States. They are one of North America’s most numerous songbirds with a population estimated at greater than 200 million birds^21^. These birds can congregate in large flocks on concentrated animal feeding operations (CAFOs), with roosts often reaching over 100,000 individuals, especially in the absence of a naturally occurring food source^22^. We have observed European starling movement between livestock production facilities, with reported banding and recovery data indicating that these birds may migrate distances exceeding 1,500 km in North America^23^. European starlings also thrive in urban landscapes, where food, water, and nesting resources are met^21^. Given these behavioral and ecological propensities, European starlings were hypothesized to play an important role in the dissemination of pathogenic bacteria, including AMR bacteria, to livestock and subsequently humans^24–27^. In our companion work, we identified that the total population of European starlings found on CAFOs was positively correlated with cattle fecal shedding of ciprofloxacin (CIP)-resistant *Escherichia coli* (*E. coli*), further highlighting an important role of these birds in the maintenance and dissemination of AMR^28^.

The prevalence and diversity of AMR in European starlings associated with livestock operations remain largely uncharacterized, and have not been studied using high resolution genomic analyses. Based on our current knowledge of the ecology of European starlings and AMR, we hypothesized that these birds play an important role in the mechanical transmission of AMR among CAFO’s across large geographical distances, with transmission patterns displaying elements of clonality. Thus, the objective of the present study was to subject specific AMR indicator *E. coli* present within CAFO-associated European starlings to sequential analyses to characterize potential transmission dynamics. Specifically, indicator *E. coli* with phenotypic resistance to fluoroquinolones and third-generation cephalosporins (3G-C) were targeted due to the classification of these antibiotics as critical AMR priorities^4,29^. Indicators were collected from European starlings within an extensive network of 31 feedlots in five U.S. states. A suite of phenotypic and molecular analyses were performed to assess AMR and phylogenetic relationships of these isolates.

## Results

### **Occurrence of AMR*E. coli*in European starling gastrointestinal tracts**

A total of 339 presumptive *E. coli* isolates displaying resistance to subinhibitory concentrations of cefotaxime (CTX) or CIP were collected using culture-based methods from 1,477 European starling gastrointestinal tracts. These birds were obtained from feedlots located in major beef cattle production areas in the United States. From the collected isolates, 236 were confirmed as *E. coli* via matrix assisted laser desorption/ionization (MALDI) biotyping, with 60 isolates (from 59 birds) obtained from CTX-selection (prevalence of 4%) and 176 isolates (from 150 birds) obtained using CIP-selection (prevalence of 10%) (Supplementary Table 1). There were 42 birds with confirmed *E. coli* isolates obtained from both CTX- and CIP-selection. The majority of the confirmed *E. coli* isolates (*n* = 206) were collected in Kansas and Texas.

### **Extensive drug resistance in*E. coli*isolates from European starlings**

**Figure 1:**
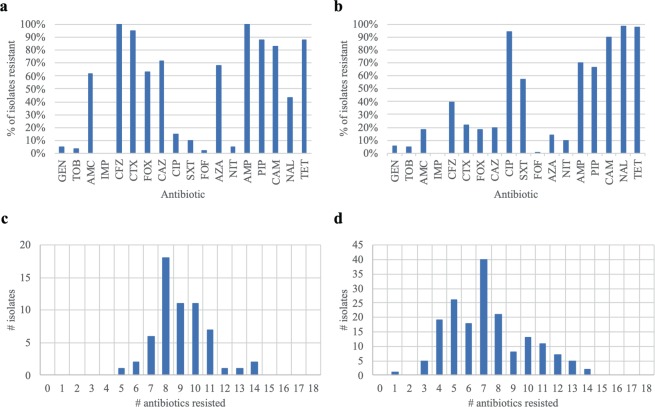
Extent and diversity of clinically significant AMR phenotypes in CTX- and CIP-selected *E. coli* isolates collected from European starlings on CAFOs. Percent of (**a**) CTX-selected and (**b**) CIP-selected *E. coli* isolates for resistance to 18 antibiotics. The number of antibiotics resisted by (c) CTX-selected and (d) CIP-selected *E. coli* isolates.

The 236 isolates confirmed as *E. coli* were subject to phenotypic assessments of antimicrobial susceptibility to 18 different antibiotics in order to 1) establish whether clinically significant resistance was present to priority antibiotics, 2) determine the diversity and extent of multidrug resistance in these isolates, and 3) compare antibiograms for subtyping (Fig. 1).

Among CTX- and CIP-selected isolates, 95% and 94% of these isolates, respectively, displayed clinically significant levels of resistance to the antibiotics used for selection. As expected, culture-based selection influenced the antimicrobial susceptibility patterns observed. CTX-selected isolates and CIP-selected isolates were generally resistant to β-lactam antibiotics and to quinolones, respectively (Figs. 1a and 1b). More than 88% of the isolates were resistant to tetracycline (TET). A greater proportion of CIP-selected isolates (57% of isolates) were resistant to sulfamethoxazole/trimethoprim (SXT) compared to isolates selected with CTX (10% of isolates). None of the isolates tested here were found to have resistance to carbapenems (i.e. imipenem; IMP). On average, CTX-selected *E. coli* isolates were resistant to 9 of the antibiotics tested, whereas the CIP-selected *E. coli* were resistant to 7.3 antibiotics (Figs. 1c and 1d). Additionally, these isolates were resistant to an average of 6 different classes of antibiotics (see Supplementary Table 1 for the antibiotic classes tested). Phenotypic testing allowed for grouping of these isolates into 42 different antibiograms with unique AMR phenotypes to β-lactams (Supplementary Table 2). The antibiograms comprised between 1 and 29 isolates, which displayed resistances to as few as one β-lactam (ampicillin; AMP) to as many as 8 β-lactams (all β-lactams tested excluding IPM).

### **PCR-based characterizations of β-lactamase genes and determinations of*E. coli*phylotypes**

Confirmed *E. coli* isolates (*n* = 187) which demonstrated resistance to any of the nine β-lactam antimicrobials included in the testing panel were further typed by conventional PCR to screen for several major class A β-lactamase genes. β-lactamase genes identified included *bla*_AmpC-CIT_ (*n* = 56 isolates), *bla*_CTX-M_ (*n* = 35 isolates), and *bla*_TEM_ (*n* = 90 isolates) (Supplementary Table 2).

The presence of *bla*_AmpC-CIT_ was identified in isolates exhibiting a broad spectrum of resistance, often conferring resistance to all β-lactam antimicrobials tested (except IPM). Such relationships between the presence of *bla*_AmpC-CIT_ and the β-lactam resistance phenotypes observed is highlighted in isolates belonging to antibiograms # 8, 20, and 22. The presence of *bla*_TEM_ was determined in the isolates which were more frequently comprised in antibiograms # 3, 4, 5, 6, 34, 35, 40, and 41, and primarily conferred resistance to penicillins (AMP, piperacillin; PIP) or intermediate resistance to cefazolin (CFZ) and amoxicillin-clavulanate (AMC). *bla*_CTX-M_ was detected primarily in isolates grouped within antibiograms # 11, 16, 17, 25, and 27, and was mostly linked to AMR involving increased resistance to first-generation (1G-C) and second-generation (2G-C) cephalosporins. The association of TEM-1 and TEM-2 with increased hydrolysis of penicillins and 1G-C, and that of *bla*_CTX-M_ (*bla*_CTX-M-15_) with increased hydrolysis of other β-lactams and monobactams is well established^30^.

PCR-based phylotyping classified 29, 120, 36, 1, and 1 isolates as *E. coli* phylotypes A, B1, D, B2, and C, respectively. Often, the presence of *bla*_AmpC-CIT_ (CIT-type AmpC β-lactamases, including *bla*_CMY-2,-3,-4_, *bla*_LAT-1_, *bla*_LAT-2_, *bla*_BIL-1_) was found in isolates from phylogroups B1 and D, and the presence of *bla*_TEM_ in isolates from phylogroups A and B1, and *bla*_CTX-M_ was associated with phylogroups B1 and D (Supplementary Table 2).

### **Genetic context of AMR in*E. coli*collected from European starlings**

 A prioritized subset of 66 isolates was selected for whole genome sequencing (WGS). *In silico* analyses of the partial genomes identified a total of 3,435 AMR genetic determinants (94 unique putative AMR genes), with between 35 and 61 unique genes identified per isolate. The majority of the putative AMR genes were associated with drug efflux, and numerous genes involved in antibiotic inactivation and target alteration were identified (Table 1).

**Table 1:** Number and type of AMR determinants predicted in 66 priority *E. coli* isolates collected from European starlings.

Mechanism of action	# determinants
antibiotic efflux	2256
antibiotic efflux; antibiotic target alteration	198
antibiotic inactivation	276
antibiotic target alteration	461
antibiotic target alteration; antibiotic efflux	5
antibiotic target protection	39
antibiotic target replacement	69
reduced permeability to antibiotic; antibiotic efflux	66
reduced permeability to antibiotic; antibiotic efflux; antibiotic target alteration	65

**Figure 2:**
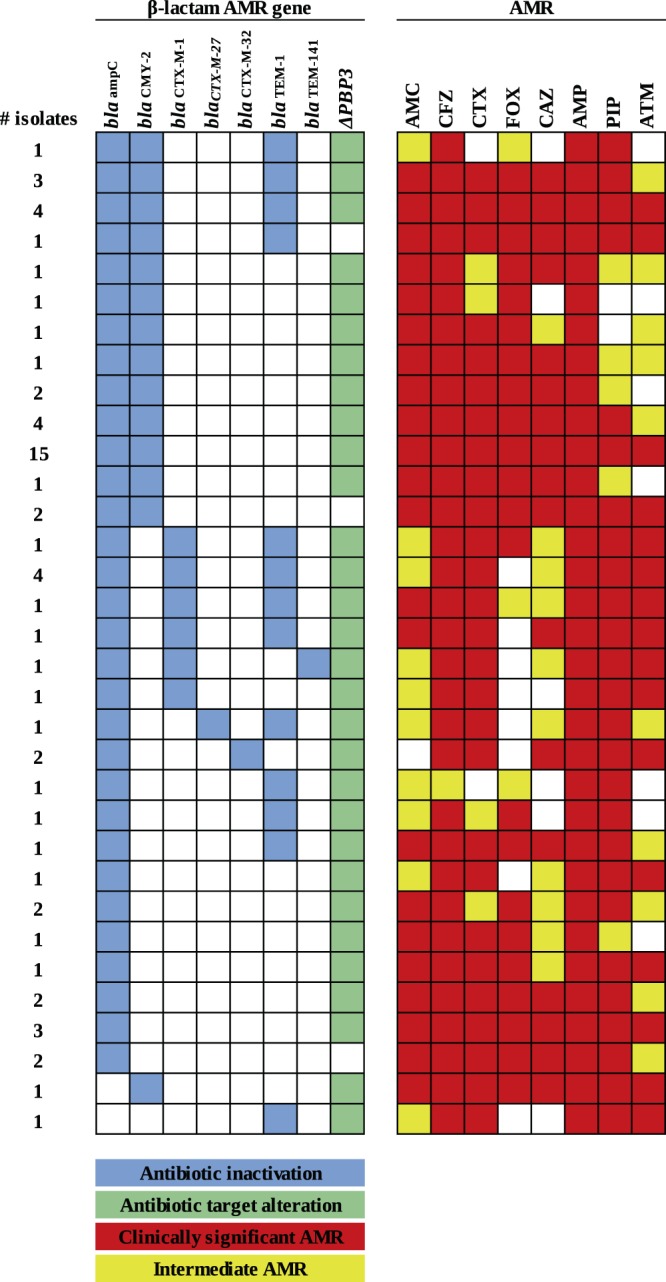
*In silico* assessments of AMR genes found within 66 *E. coli* isolates collected from European starlings that were predicted to confer resistance to β-lactams. CARD-RGI analyses of isolates identified a total of 8 genes which putatively confer resistance to β-lactams. An additional 12 genes putatively involved in efflux of both β-lactams and quinolones were identified (see Supplementary Table 3). The column labeled ‘AMR’ represents phenotypic resistance characterization, and it is matched by row to the genotypic data presented in the left side columns. Cells shaded in white indicate that the gene was not present or the isolate was susceptible to the particular drug.

**Figure 3:**
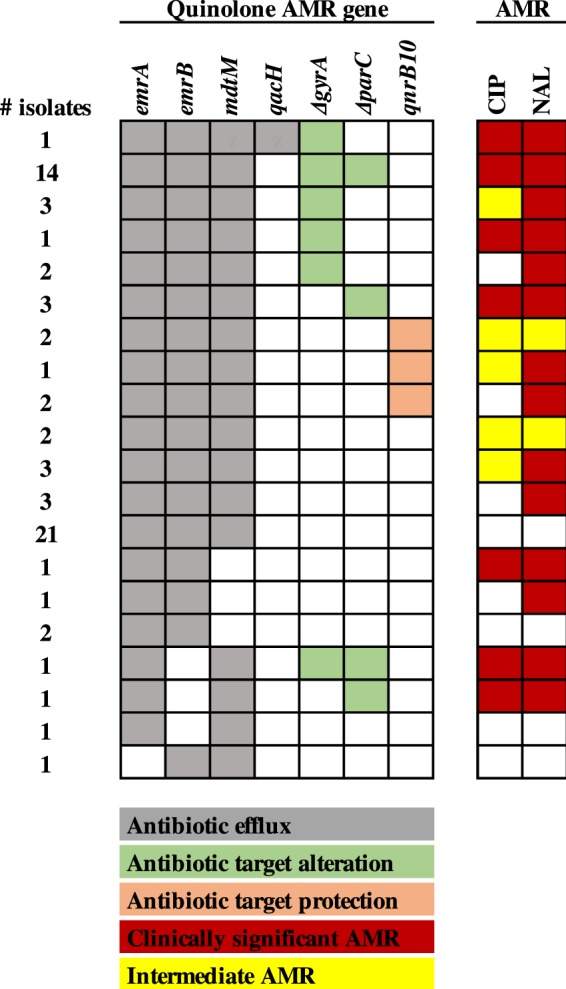
*In silico* assessments of AMR genes found within 66 *E. coli* isolates collected from European starlings that were predicted to confer resistance to quinolones. CARD-RGI analyses of isolates identified 7 genes that putatively confer resistance to quinolones. An additional 12 genes putatively involved in efflux of both β-lactams and quinolones were identified (see Supplementary Table 3). The column labeled ‘AMR’ represents phenotypic resistance characterization, and it is matched by row to the genotypic data presented in the left side columns. Cells shaded in white indicate that the gene was not present or the isolate was susceptible to the particular drug.

Bioinformatic analyses of isolate genomes identified a total of 81 unique genetic resistance determinants that were differentially present or absent among the *E. coli* strains tested (Supplementary Table 3). Focusing these analyses on priority AMR phenotypes, seven genes putatively involved in β-lactam resistance, including *bla*_ampC_ (*n* = 64 isolates), *bla*_CMY-2_ (*n* = 38 isolates), *bla*_CTX-M-1_ (*n* = 9 isolates), *bla*_CTX-M-27_ (*n* = 1 isolate), *bla*_CTX-M-32_ (*n* = 2 isolates), *bla*_TEM-1_ (*n* = 21 isolates), and *bla*_TEM-141_ (*n* = 1 isolate) were identified (Fig. 2). Further, seven genes specifically associated to quinolone resistance were detected, including *emrA* (*n* = 65 isolates), *emrB* (*n* = 63 isolates), mutations of *gyrA* (*n* = 22 isolates), *mdtM* (*n* = 62 isolates), mutations of *parC* (*n* = 19 isolates), *qacH* (*n* = 1 isolate), and *qnrB10* (*n* = 5 isolates) (Fig. 3). Resistance to quinolones and fluoroquinolones was generally observed in isolates with mutations in *gyrA* and *parC* which are widely recognized for their ability to confer increased resistance to these antibiotics. It is important to note that the associations of several of the AMR genes identified in these homology-based searches are only weakly linked to phenotypic resistance, including the observed mutations and variants of *PBP3*, *emrA*, *emrB*, *mdtM*, *qacH*, and *qnrB10*. In addition, 12 determinants potentially associated with antibiotic efflux, and subsequent AMR to β-lactams and quinolones, were detected (Supplementary Table 3).

Analyses of the predicted protein structures indicated that *bla*_CMY-2_, *bla*_CTX-M-1_, and *bla*_CTX-M-32_ were conserved among the isolates tested here. Two distinct sequence variants of *bla*_TEM-1_ were present, with three isolates having an N-terminal truncation, which removed the first 28 amino acids compared to the other isolates (sequences otherwise identical except for V29 to M29 in isolates with the truncated determinant). Structural variability in *bla*_amp_ genes was linked to both a conserved N-terminal truncation as well as amino acid substitutions at 35 different sites.

### **Phylogenetic relationships and molecular epidemiology of AMR*E. coli*collected from European starlings**

**Figure 4:**
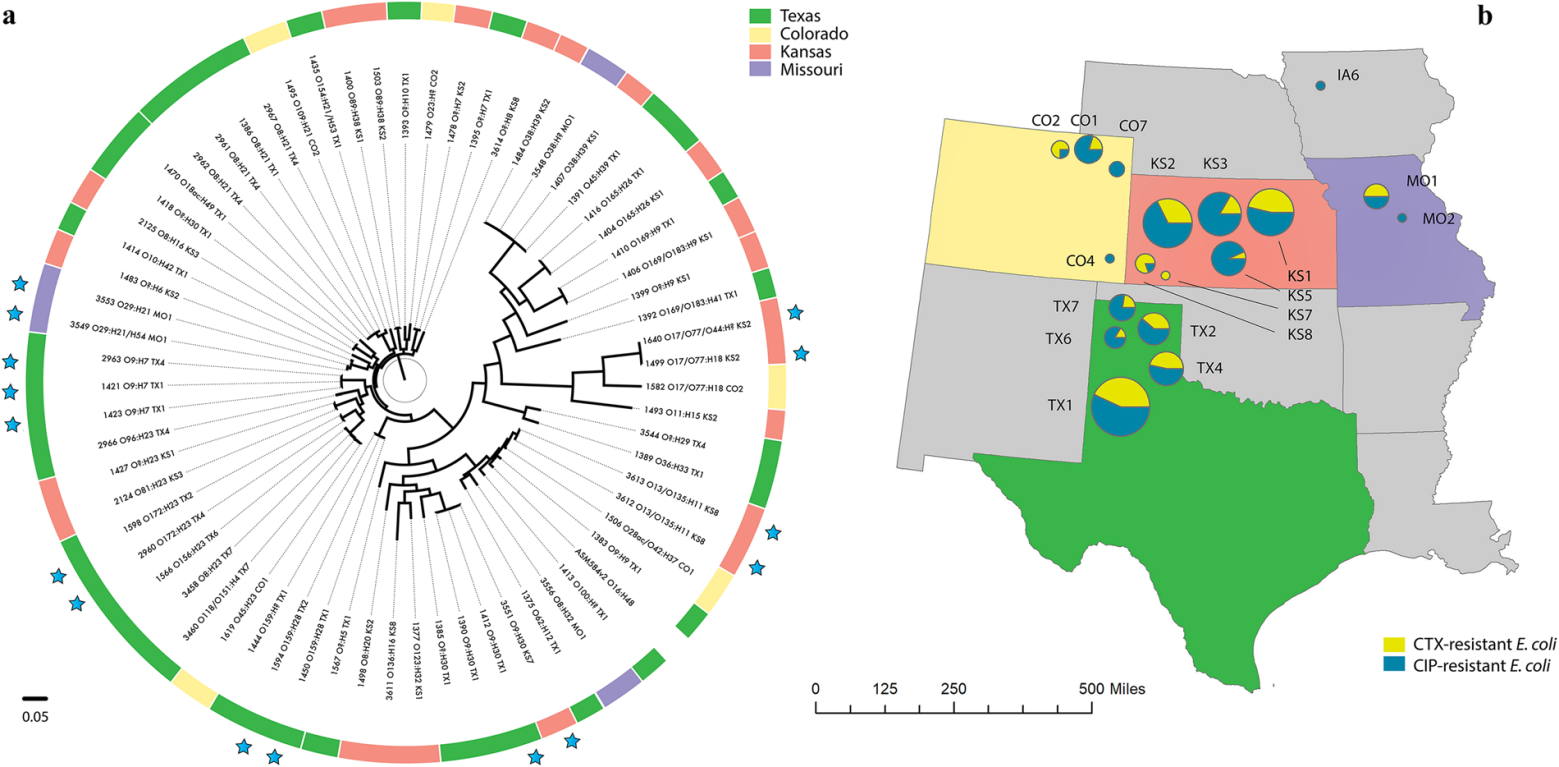
(**a**) Phylogenetic analysis of the core genomes of 66 *E. coli* isolates from European starlings. The color-coded outer ring shows the U.S. state in which the individual isolates were collected. Isolates are labelled by their sample ID #, predicted serotype, and location (state and CAFO ID). Colored stars marked at adjacent branches indicate isolate groups showing high sequence and coding region similarity (see Supplementary Table 4). (**b**) Map displaying an approximate location of CAFOs, and the proportion of all 236 CTX-resistant (yellow) or CIP-resistant (blue) *E. coli* isolates collected from these sites, with circle size proportionate to the total number of isolates collected from these sites.

Phylogenetic analysis of the 66 sequenced *E. coli* genomes showed that the core genomes of the isolates did not always align with isolates within respective collection sites, rather several isolates showed higher similarity with disparate sites across the four states represented in sequenced samples (Fig. 4). Geographically separate isolate groups displayed up to 99.9% similarity at the nucleotide level and up to 100% in shared coding sequences (CDS; Supplementary Table 4) across their assembled genomes and exhibited identical or nearly identical AMR phenotypes and molecular features predicted *in silico* (Supplementary Tables 2 and 3). Four of the isolate groups were collected on farms separated by 21–183 km (Supplementary Table 4).

Predictions of serotypes *in silico* were unambiguous for 40 of the *E. coli* isolates, with 28 unique serotypes detected (Supplementary Table 5). According to the Enterobase database (https://enterobase.warwick.ac.uk/species/index/ecoli, accessed 10/2/19), serotypes identified here, including O109:H21, O11:H15, O159:H28, O29:H21, O8:H21, and O9:H7, were previously associated with disease in humans, and a number of these *E. coli* serotypes were previously found in cattle (including O10:H42, O136:H16, and O9:H9), food products including poultry and leafy greens (including O11:H15, O136:H16, O18ac:H49, O8:H16, and O8:H20), and wildlife (including O10:H42, O109:H21, O11:H15, O172:H23, and O8:H21).

Two multilocus sequence typing schemes were used for further subtyping assessment of the *E. coli* isolates (Supplementary Table 5), with 23 and 18 clonal groups detected with high confidence, respectively. According to the Enterobase database, the majority of the *E. coli* sequence types (ST) identified here are recognized to occur in livestock, food, wildlife, as well as the environment and were previously shown to have priority AMR phenotypes/genotypes. *E. coli* isolates 1567 (ST4542) and 2966 (ST4380) had exceedingly rare STs, with less than 10 entries for these STs described in the Enterobase database. These STs were previously observed in poultry-associated isolates from China, and are primarily recognized in Asia and the Philippines, although ST4380 was also detected in a *bla*_CTX-M-55_-carrying *E. coli* isolate collected from a monkey in France in 2011^31^.

## Discussion

In this study, we examined the AMR phenotypes and molecular epidemiology of CTX and CIP-resistant *E. coli* at the interface of livestock and European starlings. Importantly, we provide evidence for genetically conserved AMR isolates in European starlings separated by distances exceeding 150 km on cattle production facilities in the Central United States. While directionality of the exchange of these bacteria could not be specifically inferred, our data strongly suggest that European starlings are involved in interstate dissemination of specific lineages of bacteria with priority AMR phenotypes. Our future research efforts will determine if AMR strains harbored by these birds can become established in cattle and CAFO environments.

The identification of CIP- and CTX-resistant *E. coli* in European starlings from CAFOs was not altogether unexpected, as AMR in the microbial communities associated with livestock production is widespread and is linked to a diverse array of phenotypic and genetic determinants^32^. The observation that CTX-selected isolates were resistant to a greater number of antibiotics was also not surprising  considering the types of antibiotics included in the testing panel. Specifically, nine different β-lactams were employed in susceptibility testing, more than any other type of antimicrobial. Further, AMR in CTX-selected isolates was mediated by genetic determinants which typically conferred resistance to a broad range of antimicrobials.

The dynamics of AMR dissemination and introduction into agricultural systems are difficult to interpret considering the number of events and sources in which AMR bacteria could be introduced and disseminated. Nonetheless, based on current research, a logical assumption is that competent wildlife hosts that are found in high numbers on livestock facilities and can travel over large distances would be particularly important contributors to AMR in these settings. High resolution analyses of the genetic backgrounds of such a diversity of *E. coli* isolates obtained from European starlings had not been investigated previously. This information is critical for assessing the potential role of European starlings as contributors to AMR in livestock production. Molecular typing revealed striking similarities between serotypes and STs from European starlings and isolates from food animals, food, humans, and the environment. This information, in conjunction with the high degree of genomic conservation between certain isolates found in European starlings, provides evidence that clonal spread of AMR *E. coli* may be mediated by these birds. Similarly, a role of European starlings in the clonal dissemination of the foodborne bacterium *Campylobacter jejuni* in cattle operations has been previously suggested^33^, even though the analysis of evolutionary relationships of *E. coli* using molecular typing more closely aligns with reticulate evolution as opposed to clonality^34^. Further, evidence points to presence of multi-resistant *E. coli* clones (i.e. ST131, ST69, ST23) in animals (companion and food animals), foods, and the environment, indicating complex transmission patterns and wide distribution^35^. In our work, we observed no preponderance of specific STs, although certain patterns have emerged, as for example the identification of ST10 in several isolates associated with Inc-type plasmids, with this ST linked by others to both production of ESBL enzymes^36^ and hyperexpression of AmpCs^37^.

Multiple studies have suggested that interactions between CAFO cattle and wildlife, including European starlings, contribute to increased cattle infection/fecal shedding of AMR bacteria, and environmental dissemination of AMR bacteria^22,24,38–40^. This risk is further highlighted in a study by Medhanie *et al*. which suggested that increased prevalence of CTX and CIP-resistant *E. coli* in bovine feces correlated with increased populations of European starlings, and that the distance between livestock facilities and European starling roosts was significantly associated with the presence of these AMR indicators^41^. A similar result was achieved in the companion study to this work, which indicated that shedding of CIP-resistant *E. coli* in cattle is positively correlated with a greater abundance of European starlings utilizing the associated production environments^28^.

In assessments of β-lactam resistance among the isolates collected in this study, the presence of *bla*_AmpC-CIT_ was often found in conjunction with broad-spectrum resistance to β-lactams, including 3G-C antimicrobials such as CTX and CAZ, as well as monobactams represented by ATM. Resistance phenotypes involving reduced susceptibility to 3G-C, and monobactams are commonly mediated by extended-spectrum and AmpC β-lactamases^42,43^. CMY-2 is considered to be the most common type of plasmid-mediated AmpC β-lactamase, frequently isolated from patients in hospitals, livestock, and ground meat^44–46^. It should be noted that the majority of isolates characterized in our study, including those containing presumptive plasmid-mediated AmpCs, were determined to belong to the phylogenetic group B1. This phylogroup comprises primarily commensal bacteria^7^, which have been shown to resist stress and persist better in the environment^47^. Additionally, *E. coli* phylogenetic group B1 was previously found in European starlings sampled in Ireland^24^, providing an indication of host preferences for this phylogroup. PCR, which we utilized as a screening tool for select antimicrobial resistance genotypes, was generally in excellent agreement with outcomes of WGS characterization. When discrepancies occurred, these were primarily due to incomplete coverage of the primer sets utilized, or a limitation associated with short-read sequencing, which generally results in partial genomes.

A large percentage of the isolates in our study were resistant to TET and CAM. To date, TET resistance remains one of the most commonly encountered resistances in *E. coli*^48^. TET resistance has been associated with decreased susceptibility to SXT and AMP in *E. coli* from food animals in multiple European countries. The resistance genetic determinants to these antimicrobials are often found on the same mobile genetic elements, potentially reflecting the history of antimicrobial use in these countries^49^. The high percentages of resistance to CAM in our isolates were somewhat unusual. Only two of the isolates sequenced here encoded specific exporters recognized to confer resistance to phenicols (60 of the 66 isolates sequenced had clinical-resistance to CAM). However, several mechanisms are associated with CAM resistance in bacteria^50^, including target site mutations or modifications^51^, acetylation via acetyltransferases or chloramphenicol phosphotransferases^52,53^, efflux pumps^54^, and decreased outer membrane permeability^55^. Additionally, we note that CAM is banned for use in veterinary medicine (including food animals) in the United States due to issues related to its toxicity^56,57^. Nevertheless, florfenicol (fluorinated synthetic analog of thiamphenicol, a CAM analog) is used to treat respiratory infections in cattle and resistance to this antibiotic is documented in livestock and may be co-selective^58,59^.

Here, we assessed the phenotypic and genetic diversity of AMR *E. coli* isolates collected from European starlings found on CAFOs in order to improve our understanding of the role of these birds in the transmission of AMR in livestock production systems. We utilized an array of methods, ranging from culture-based to molecular screening to select for priority phenotypes and genotypes for advanced molecular typing and characterization. We selected priority phenotypes and genotypes for downstream WGS analysis in order to facilitate phylogenetic and AMR determinant characterization; however, this approach would not be considered optimal should the goals of the study benefit from determination of genomic diversity in all isolates.

This study shows that *E. coli* isolated from these birds have important AMR phenotypes and genes, including those which confer resistance to fluoroquinolones and β-lactam antimicrobials, including 3G-C. Our study also identified phylogenetically conserved *E. coli* isolates in geographically separated European starlings, highlighting a potential link between this invasive agricultural pest and interstate dissemination of AMR in food animal production.

## Materials and Methods

### Study sites, collection of avian fecal specimens, and culture-based isolation

Feedlots with severe European starling problems (e.g., experiencing more than 10,000 European starlings per day) were previously identified using methodology established for identifying bird damage associated with dairies^60^. Samples from 31 feedlots in Colorado (*n* = 8 feedlots, 400 birds), Iowa (*n* = 5 feedlots, 150 birds), Kansas (*n* = 8 feedlots, 443 birds), Missouri (*n* = 3 feedlots, 119 birds), and Texas (*n* = 7 feedlots, 365 birds) were collected between December 4, 2012 and March 12, 2013. Up to 30 European starlings were collected at each livestock facility per day following methods approved by the United States Department of Agriculture (USDA) National Wildlife Research Center’s (NWRC) Animal Care and Use Committee. European starling collections were conducted using shotguns as set forth by agency policy in the USDA/Animal and Plant Health Inspection Service (APHIS)/Wildlife Services (WS) Directive 2.505. Identifying information was recorded for each bird collected, including an assigned facility number, time and date of collection, and location of collection. Bird carcasses were individually bagged in sterile Whirl-Pak bags (Nasco, Fort Atkinson, WI) and shipped overnight at 4 °C to our laboratories for processing.

### Culture-based isolation

The gastrointestinal tract, from proventriculus to cloaca, was removed from each European starling, placed into a sterile Whirl-Pak bag (Nasco, Fort Atkinson, WI), and homogenized for 120 sec at 230 rpm using a Stomacher 400 Circulator (Seward, Islandia, NY). The resulting homogenate was inoculated using a sterile cotton tipped applicator onto MacConkey agar (Acumedia, Lansing, MI) (MAC) supplemented with 2 μg/mL CTX (Calbiochem, EMD Millipore, Billerica, MA) and onto MAC supplemented with 1 μg/mL CIP (Enzo Life Sciences, Farmingdale, NY) and incubated at 37 °C for 24 hr. From each media, 1–2 presumptive *E. coli* colonies (occasionally lactose negative isolates were also collected) were subcultured on either MAC-CTX or MAC-CIP to yield purified isolates. Purified isolates were propagated overnight in Brain Heart Infusion Broth (BD, Franklin Lakes, NJ) at 37 °C with shaking at 200 rpm, then mixed 1:1 in 40% glycerol, and stored at −80 °C until further use.

### Isolate confirmation

Species-level confirmation was performed on all bacterial isolates presumptively identified as *E. coli* via culture-based methods by MALDI biotyping using a formic acid-acetonitrile extraction procedure^61^. Briefly, one loopful (approximate volume of 1 μl) of bacteria was suspended in a 1:3 solution of HPLC grade water (Sigma-Aldrich, St. Louis, MO) and absolute ethanol (Sigma-Aldrich). According to the manufacturer’s protocol, bacteria were collected by centrifugation at 17,000 × *g*, pellets were allowed to air dry, and then suspended 1:1 in acetonitrile (Sigma-Aldrich, St. Louis, MO) and 70% formic acid (Sigma-Aldrich, St. Louis, MO). Insoluble material was pelleted by centrifugation as described above and 1 μl of the supernatant was applied to a polished steel target plate (Bruker, Billerica, MA). Samples were air-dried and overlaid with 1 μl of freshly prepared α-cyano-4-hydroxycinnamic acid (Bruker, Billerica, MA). MALDI biotyping for genus and species identification was accomplished using a Bruker Ultraflex II TOF/TOF or Bruker Microflex LRF (Billerica, MA) operating with Bruker Biotyper RTC software (Version 3.1) and pre-calibrated with Bruker Bacterial Test Standard. Species level identification of the isolates was accepted if a score of ≥1.7 was assigned by the MALDI Biotyper algorithm. While species-level identification is not secure with a score of ≤2.0, a lower score (≥1.7) was accepted when supported by culture-based identification.

### Antimicrobial susceptibility testing

Antimicrobial susceptibility testing of *E. coli* isolates was performed in accordance with Clinical and Laboratory Standards Institute’s (CLSI) M100-S24 protocols^62^. Sensi-Discs impregnated with the following antibiotics were used: gentamicin (GEN), tobramycin (TOB), AMC, IPM, CFZ, CTX, FOX, ceftazidime (CAZ), CIP, SXT, fosfomycin (FOF), ATM, nitrofurantoin (NIT), AMP, PIP, CAM, nalidixic acid (NAL), and TET. Antimicrobial susceptibilities were classified as sensitive, intermediate, or resistant based on the measured zones of inhibition and CSLI-established cutoffs^62^.

### PCR-based phylotyping and detection of select AMR genes conferring β-lactam resistance

Phylotype grouping (Supplementary Table 6) was performed using a triplex PCR as previously described by Clermont *et al*.^63^. Briefly, all DNA was prepared via conventional boil-prep. The conventional PCR assay targeted *chuA*, *yjaA* and an anonymous DNA fragment (*tspE.C2*). An Applied Biosystems 2720 thermal cycler (Foster City, CA) was used for amplification. This procedure permitted classification of the isolates into phylogroups. The class A ß-lactamase genes *bla*_CTX-M_, *bla*_SHV_, *bla*_TEM_ and CIT-type AmpCs were detected in a one-step multiplex PCR reaction as previously described by Roschanski *et al*. using a CFX96 Touch Real-Time PCR Detection System (Bio-Rad laboratories, Hercules, CA, USA) (Supplementary Table 6)^46^. All primers and probes were from Integrated DNA Technologies, Inc. (Coralville, IA).

### Whole genome sequencing and analysis

The epidemiological, phenotypic, and PCR data collected were used to prioritize a subset of isolates for genetic analyses by WGS. Priority was given to *E. coli* isolates with similar AMR phenotypes collected between multiple sampling locations and with clinically significant 3G-C resistance (*n* = 66).

DNA libraries were prepared using the Nextera XT chemistry with Nextera XT version 2 indexes (Illumina, San Diego, CA, USA). The DNA from each isolate was subject to bead-based size exclusion to optimize DNA fragment size in accordance with standard procedures^64^. Using the Illumina NextSeq with 150 bp paired-end reads, 5 ng of each sample were pooled and analyzed. The resultant reads were scanned for contamination with Kraken^65^, trimmed for quality using Trimmomatic (ILLUMINACLIP:BBmapAdapters.fasta:2:30:10 HEADCROP:10 SLIDINGWINDOW:4:24 MINLEN:100)^66^, and assembled *via* SPAdes v3.13 (assemble only, kmer values 21, 33, 55, 77)^67^. Structural and functional annotation of the resultant scaffolds was achieved with Prokka v1.13 (Genera: *Escherichia*)^68^. Unrooted core genome phylogeny comparisons were completed using Parsnp (with GenBank accession ASM584v2 as the reference genome) and visualized in FigTree v1.4.4 (http://tree.bio.ed.ac.uk/software/figtree)^69–72^. For assembly metrics see Supplementary Table 7^73^. Highly similar isolate groups were further interrogated *via* Spine v0.3.1 (core assignment must occur in all genomes, minimum percent identity of alignments at the nucleotide level = 75, minimum core assignment size = 200 bp, maximum distance between core genome segments = 10 bp, see Supplementary Table 4)^74–76^. The Resistance Gene Identifier tool and Comprehensive Antibiotic Resistance Database (web version) run on the Prokka amino acid predications were used to identify determinants putatively involved in antibiotic resistance^77^. Additional subtyping analyses for serotype predictions, multilocus sequence typing, *fimH* type, *fumC* type, and plasmid identification were performed using tools available at the Center for Genomic Epidemiology (http://www.genomicepidemiology.org/)^78–80^. *E. coli* STs were further evaluated using the Enterobase database (https://enterobase.warwick.ac.uk/species/index/ecoli, accessed 10/2/19).

## Supplementary information

Supplementary information.

Supplementary information2.

Supplementary information3.

Supplementary information4.

Supplementary information5.

Supplementary information6.

Supplementary information7.

Supplementary information8.
