# Author Correction: The Role of European Starlings (*Sturnus vulgaris*) in the Dissemination of Multidrug-Resistant *Escherichia coli* among Concentrated Animal Feeding Operations

**DOI:** 10.1038/s41598-020-68365-9

**Published:** 2020-07-15

**Authors:** Jeffrey C. Chandler, Jennifer E. Anders, Nicolas A. Blouin, James C. Carlson, Jeffrey T. LeJeune, Lawrence D. Goodridge, Baolin Wang, Leslie A. Day, Anna M. Mangan, Dustin A. Reid, Shannon M. Coleman, Matthew W. Hopken, Bledar Bisha

**Affiliations:** 1 U.S. Department of Agriculture, National Wildlife Research Center, Fort Collins, CO USA; 2 Department of Animal Science, University of Wyoming, Laramie, WY USA; 3 Department of Molecular Biology, University of Wyoming, Laramie, WY USA; 4 Food and Agriculture Organization of the United Nations, Rome, Italy; 5 Food Science Department, University of Guelph, Guelph, ON Canada; 6 Department of Food Science and Human Nutrition, Iowa State University, Ames, IA USA; 7 Department of Microbiology, Immunology, and Pathology, Colorado State University, Fort Collins, CO USA

Correction to: *Scientific Reports* 10.1038/s41598-020-64544-w, published online 15 May 2020

The Supplementary information5 originally published with this Article contained redundant data. This redundant data has been removed from the Supplementary Information5 that now accompanies the Article.

