# Supplementary material for: The Role of European Starlings (Sturnus vulgaris) in the Dissemination of Multidrug-Resistant Escherichia coli among Concentrated Animal Feeding Operations
Source: Sci Rep. 2020 May 15;10:8093. doi: 10.1038/s41598-020-64544-w (PMC7229194; doi:10.1038/s41598-020-64544-w)
Supplement: Supplementary file 8 — Supplementary information8. [file 41598_2020_64544_MOESM8_ESM.docx]

**Title:** The Role of European Starlings (*Sturnus vulgaris*) in the Dissemination of Multidrug-Resistant *Escherichia coli* among Concentrated Animal Feeding Operations

**Running Title:** AMR bacteria in European starlings linked to livestock

**Authors:** Jeffrey C. Chandler^a^, Jennifer E. Anders^b^, Nicolas A. Blouin^c^, James C. Carlson^a^, Jeffrey T. LeJeune^d^, Lawrence D. Goodridge^e^, Baolin Wang^b^, Leslie A. Day^b^, Anna M. Mangan^a^, Dustin A. Reid^a^, Shannon M. Coleman^f^, Matthew W. Hopken^g^, and Bledar Bisha^b^*

**Affiliations:**

^a^U.S. Department of Agriculture, National Wildlife Research Center, Fort Collins, CO. USA. ^b^University of Wyoming, Department of Animal Science, Laramie, WY, USA. ^c^University of Wyoming, Department of Molecular Biology, Laramie, WY, USA.

^d^Food and Agriculture Organization of the United Nations, Rome, ITA.

^e^University of Guelph, Food Science Department, Guelph, Ontario, CAN.

^f^Iowa State University, Department of Food Science and Human Nutrition, Ames, IA, USA.

^g^Colorado State University, Department of Microbiology, Immunology, and Pathology, Fort Collins, CO. USA.

**Corresponding Author:**

Bledar Bisha

Mailing address:

Department of Animal Science, University of Wyoming, 1000 E. University Ave. Dept. 3684, Laramie, WY, 82071-2000,

Phone: (307) 766-3140

Fax: (307) 766-2355

Email: [bbisha@uwyo.edu](mailto:bbisha@uwyo.edu)

**Supplementary Tables:**

**Table S1** *E. coli* isolates collected from European starlings on CAFOs from CTX- and CIP-selection, and associated epidemiological and antimicrobial susceptibility data.

**Table S2** Phenotypic and genotypic profiles of *E. coli* isolates with resistance to β-lactam antibiotics.

**Table S3** Differentially present or absent AMR genes (predicted by CARD-RGI) in 66 *E. coli* isolates derived from European starlings on CAFOs, and associated AMR phenotypes.

**Table S4.** Genome and coding sequence similarity of highly similar isolate groups identified from phylogenetic analysis.

**Table S5.** Molecular typing of *E. coli* isolates collected from European starlings.

**Table S6** Primers and probes used for PCR-based *E. coli* phylotyping and detection of β-lactamase genes.

**Table S7** WGS assembly statistics.
